# How effective is incidental learning of the shape of probability distributions?

**DOI:** 10.1098/rsos.170270

**Published:** 2017-08-02

**Authors:** Randy Tran, Edward Vul, Harold Pashler

**Affiliations:** Department of Psychology, University of California, San Diego, 9500 Gilman Drive, La Jolla, CA 92093, USA

**Keywords:** implicit learning, generative models, probability learning

## Abstract

The idea that people learn detailed probabilistic generative models of the environments they interact with is intuitively appealing, and has received support from recent studies of implicit knowledge acquired in daily life. The goal of this study was to see whether people efficiently induce a probability distribution based upon incidental exposure to an unknown generative process. Subjects played a ‘whack-a-mole’ game in which they attempted to click on objects appearing briefly, one at a time on the screen. Horizontal positions of the objects were generated from a bimodal distribution. After 180 plays of the game, subjects were unexpectedly asked to generate another 180 target positions of their own from the same distribution. Their responses did not even show a bimodal distribution, much less an accurate one (Experiment 1). The same was true for a pre-announced test (Experiment 2). On the other hand, a more extreme bimodality with zero density in a middle region did produce some distributional learning (Experiment 3), perhaps reflecting conscious hypothesis testing. We discuss the challenge this poses to the idea of efficient accurate distributional learning.

## 1. Introduction

People often seem to behave effectively based on noisy observations of uncertain environments. This might seem surprising because people generally have poor incidental memory (e.g. the direction that Lincoln faces on the penny; [1]). On the other hand, probability distributions may be special, and there is evidence that people are quite good at estimating frequencies of events even when they have paid little attention to the stimuli as they appeared [2]. One currently popular interpretation of this adaptive flexibility assumes that people efficiently learn probabilistic generative models of their environment and then use these models to guide their behaviour. Such a capability would seem to have the potential to assist people in achieving many of their goals, including goals with strong benefits to Darwinian fitness (such as finding food and finding mates). If one looks at the literature, however, while there are many examples of evidence taken to favour the idea of flexible induction of generative models, the evidence appears somewhat restricted and indirect. For example, Vulkan [3] showed that people were able to match reward probabilities of several alternatives with their choices, indicating that they can learn probability distributions over those alternatives. People can also learn the probabilistic dependency structure in networks of binary variables (e.g. [4,5]). As impressive as these feats are, these outcomes could potentially be achieved by learning only the first few moments (mean and variance) of a distribution rather than the full underlying structure. In this paper, we ask more straightforwardly: can people learn the overall shape of an observed distribution and are they able generate new instances that retain the properties of the learned distribution?

## 2. Prior methodological procedures used in distributional learning studies

To date, studies that have shown evidence for (e.g. [6,7]) and against (e.g. [8]) distributional learning have used tasks that: (i) employ other types of strategies or (ii) seem to allow for an aggregate analysis of only a few moments of the distribution rather than the whole. Our main focus is on how the methodological procedures from various studies might limit the ability to tease apart what properties are learned from a distribution.

Griffiths & Tenenbaum [6] suggested that people have acquired a great deal of information about the shape of the distribution of quantities such as *baking time for cakes, reigns of Pharaohs* and *booking time for telephone ticket booking agencies.* Their argument for this conclusion was based on participants' ability to answer questions of the form ‘If you were calling a telephone box office to book tickets and had been on hold for 3 min, what would you predict for the total time you would be on hold?’ They found that people's responses generated from their internal generative models were very similar to the true statistical distributions. However, Mozer *et al*. [9] questioned the conclusions of Griffiths & Tenenbaum [6], arguing that the excellent performance at the aggregate level might be consistent with very limited learning at the individual level (cf. [10,11]).

A broader concern with studies like Griffiths & Tenenbaum [6] is that we do not know how much exposure, and what type of exposure, people have had to events like Pharaohs and ticket-service call lines. Many of the quantities considered by Griffiths & Tenenbaum are subject to soft constraints from general world knowledge: knowing current average lifespans have increased over time, knowing that some pharaohs came into power at a very young age all impose constraints on the distribution of Pharaoh reigns. Thus, distributional knowledge about such world facts need not imply that people efficiently learn such distributions from direct observation; instead it may imply that people effectively infer this distribution as needed.

Sailor & Antoine [8] used a more controlled set of stimuli with a task requiring participants to estimate the size of squares drawn from two distributions (Experiments 1, 3 and 4: overlapping; Experiments 2 and 5: non-overlapping). On a given trial, participants were initially presented with a square drawn from one of the distributions and were coloured red or blue to distinguish which distribution the square was drawn from; however, this was never explicitly stated to the participants. Participants then had to estimate the size of the initially presented square by adjusting the size of a subsequently displayed square. Only on the last two trials of the experiment were participants asked to estimate the mean size of the red and blue squares. Sailor & Antoine found that the estimated means for both the red and blue squares did not differ from the average of the two distributional means. In other words, participants were unable to distinguish the two different distributions; instead, they grouped the red and blue squares into a single-unimodal distribution. We argue that the methodology presented by Sailor & Antoine may not well assess an individual's ability to learn the shape of a distribution, because it requires only sensitivity to averages. In a similar task, Gershman & Niv [12] had participants estimate the number of circles presented on the screen. The circles were either all red or all blue and were drawn from two different underlying distributions of quantity. In line with the findings from Sailor & Antoine, Gershman & Niv also discovered that participants' estimations were biased towards the mean of both distributions. Participants' biases, however, were reduced when the red and blue quantity distributions were further apart (i.e. more easily distinguishable; see Experiment 3 of this article for comparable results).

In a further investigation of these findings, Xu & Griffiths [7] were able to show that participants can learn properties of a bimodal distribution using a serial reproduction task. Xu & Griffiths employed a similar procedure to Sailor & Antoine [8] where participants learned to distinguish two types of fish drawn from two separate size distributions. On a given trial, a to-be-estimated fish was presented on the screen and disappeared. Participants then adjusted the size of a subsequent fish to estimate the just-seen fish. A major novelty in Xu & Griffiths’ procedure was that each estimation made by a participant was used as subsequent to-be-estimated fish. In other words, participants estimated fish sizes from their own previous estimates (i.e. a Markov chain) rather than estimating fish sizes from fish independently drawn from the experimental distribution on each trial. Using this procedure, Xu & Griffiths claimed to have demonstrated learning of a bimodal distribution. However, the argument rests on people's reconstructions of their own estimates where iterated learning can occur from trial to trial. Hence, with this paradigm, one cannot straightforwardly ask whether or not people can generate new instances that conform to a learned distribution because each trial is influenced by the previous trial.

## 3. Present study

The current study was designed to provide a test as simple and direct as possible for the idea that people implicitly learn the shape of a distribution based on observed samples of that distribution. The study represented something of a (friendly) ‘adversarial collaboration’ (cf. [13]), in that one of us (EV) was generally favourably disposed to the idea of implicit learning of generative models, while another of us (HP) was fairly sceptical of this idea, and RT at least professed neutrality.

To maximize the chances of demonstrating effective distributional learning, several features were built into the design. First, the variable whose distribution was tested was a variable that was highly relevant to actions the subjects would be performing. To arrange this, we used a ‘whack-a-mole’-type game in which the subject sought to click on an object during the brief period before it disappeared. This required paying close attention to its location as the object's sole action-relevant property. Second, we exposed subjects to a distinctive and somewhat unusual (bimodal) distribution to make it possible to test the fidelity of the distribution they learned. The test of learning used here required subjects to produce their own sequence of locations, mimicking the locations observed during the learning phase. While the virtues of this form of test can be debated (see General discussion), the goal here was to maximize the chance of finding distributional learning (see [14], for arguments that the mental representation of distributions is embodied in the ability to generate new samples from these distributions).

## 4. Experiment 1

In Experiment 1, learning was incidental: subjects played the game in Phase 1 with no expectation of being tested.

### 4.1. Method

#### 4.1.1. Participants

Thirty undergraduates at the University of California, San Diego participated in this experiment for course credit. All were naive to the purpose of the experiment.

#### 4.1.2. Distribution used in phase 1

**Figure 1.**
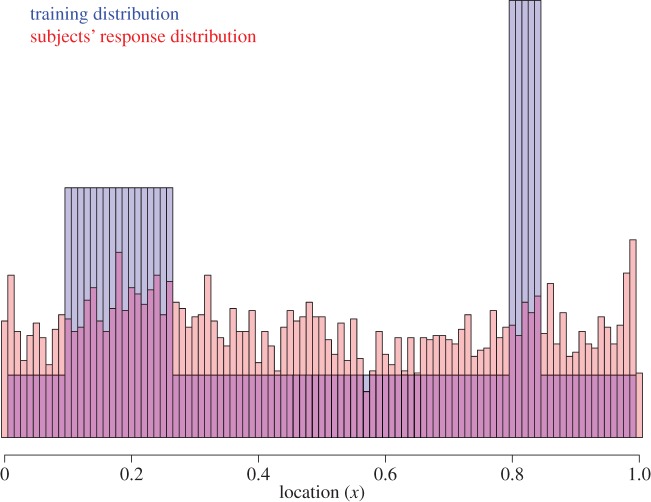
Experiment 1 results: histogram of the training (blue) and reported (red) locations.

A single fixed sequence of locations was used for all subjects in Phase 1 (the entire sequence is provided in the electronic supplementary material). The purpose of this was to avoid any confusion of the results due to sampling variability of the observations. The distribution of values used included only multiples of 0.01, with one observed value at each position within the unit interval (0, 1), plus additional values ‘piled up’ over two modes, one ranging from 0.10 to 0.26 (with four total observations at each point in that range) and the other from 0.80 to 0.84 (with seven total observations at each point in that range). Figure 1 shows this distribution.

#### 4.1.3. Procedure in phase 1

In Phase 1, subjects were told ‘Welcome to the experiment. In the first phase of the study you will play a game similar to the old computer game *Wackamole*. On every play, a disk will appear and begin expanding. Your job is simply to click on it before it disappears. If you click on it before it disappears, you score. That's it!’ They played the game 180 times. Average viewing distance from the screen was about 76 cm on a 1024 × 768-resolution screen. On each trial, a blue disc (initially just 1 pixel) appeared in a horizontal range of positions 822 pixels in width centred on the screen. Beginning at the moment of its appearance, each disc grew at 100 pixels s^−1^ until it reached a size of 100 pixels, at which time it disappeared. If the subject was able to click on the item during its 1 s expansion phase, they received 1 point. Nothing else appeared on the horizontal line. (If the subject hit the disc, a confirming sound would play with the word ‘Hit’ displayed on the screen. Otherwise a buzz sound played while the word ‘Miss’ was displayed. The feedback lasted 1.8 s. A streak counter and best streak counter were also visible on the top left of the screen, displaying the subject's current hit streak and their best hit streak overall.)

#### 4.1.4. Procedure in phase 2

Immediately after the last play, subjects began the second phase of the study, and were told, ‘Now we are interested in determining how much of an intuitive sense you have gained for how the locations of the disks were being determined. Please show us this by generating a new **sequence of locations**. Please do NOT click in the same spot over and over.’ They were also told ‘If you think there were any other patterns in the original sequence, please try to generate a sequence that reflects those patterns, too. **Don't worry about mimicking the timing** of the original sequence. Just try to produce a **sequence of locations** which is as much like the original sequence as you can make it.’

In the second phase, subjects' clicks were self-paced. When they clicked, a disc showed up with the location of the cursor as the centre of the disc, a click counter on the top left of the screen incremented with each click. After 180 clicks were registered, an exit screen was displayed, terminating the study.

### 4.2. Results and discussion

The average hit rate of clicks in Phase 1 was *M* = 0.51, s.d. = 0.14, s.e. = 0.02. Scores ranged from 0.25 to 0.74 with a median of 0.50. As with the real Whack-a-mole game, we expected to find a wide range of hit rates during Phase 1. Figure 1 shows the distribution of generated click positions aggregated across subjects for Phase 2. The subjects’ responses show no obvious similarity to the bimodal pattern presented in Phase 1.

## 5. Experiment 2

In Experiment 2, the task was the same, but the subjects were warned that they would be tested on the distributions of locations.

### 5.1. Method

#### 5.1.1. Participants

Thirty-one undergraduates drawn from the same population as Experiment 1 participated. All were naive to the purpose of the experiment.

#### 5.1.2. Materials and design

Materials and design were identical to Experiment 1 with the exception of a difference in instructions.

#### 5.1.3. Procedure

The procedure was identical to that of Experiment 1 except that prior to performing the first phase (playing Whack-a-mole), the subjects were told: ‘Just one more thing: please pay attention to the sequence of locations where the disk appears. After you're done playing, we'll ask you to try to generate a sequence of locations that simulates the sequence the computer is generating. So please see if you can learn the characteristics of the sequence of locations where the disks pop up.’

### 5.2. Results and discussion

**Figure 2.**
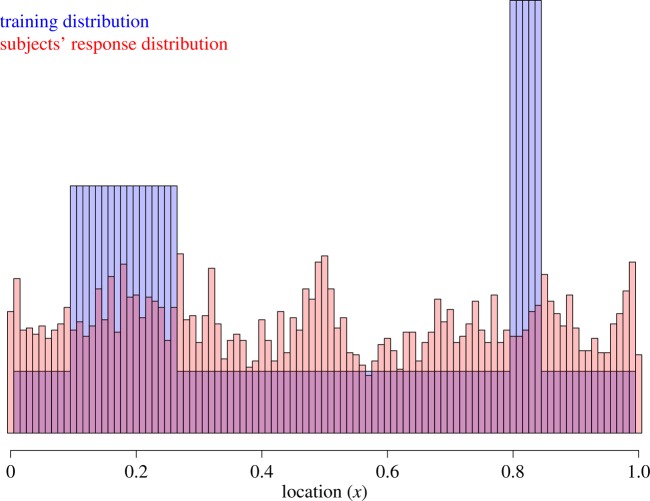
Experiment 2 results: histogram of the training (blue) and reported (red) locations.

One subject was excluded from the subsequent analyses due to a logging error in the subject's file. The average hit rate of clicks in Phase 1 was *M* = 0.57, s.d. = 0.18, s.e. = 0.03. Scores ranged from 0.17 to 0.89 with a median of 0.60. Figure 2 shows the subjects' response distribution for Phase 2. Again, there was no sign in the aggregate responses that subjects learned the bimodality of the distribution, despite an explicit instruction to try to learn the characteristics of the sequence.

## 6. Experiment 3

**Figure 3.**
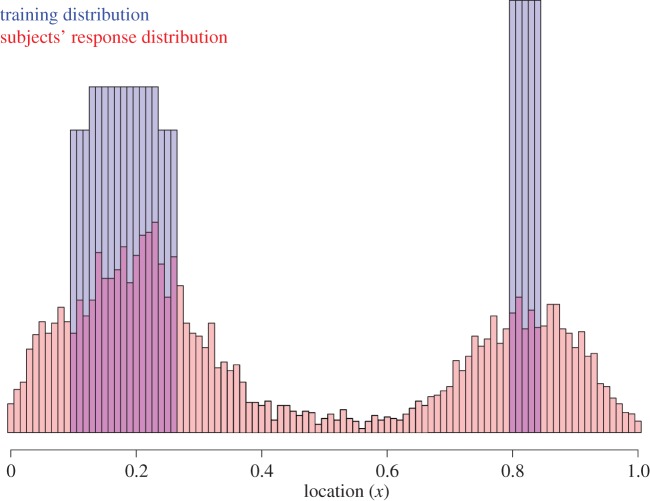
Experiment 3 results: histogram of the training (blue) and reported (red) locations.

In Experiment 3, the procedure followed Experiment 1, except the distribution used was more extremely bimodal, with zero density outside of the intervals of the modes ([0.10, 0.26] and [0.8, 0.84]; figure 3). Thirty-one undergraduates from the same subject pool participated (one subject was excluded due to a file logging error). The complete stimulus sequence is provided in the electronic supplementary material.

### 6.1. Results and discussion

The average hit rate of clicks in Phase 1 was *M* = 0.58, s.d. = 0.16, s.e. = 0.03. Scores ranged from 0.24 to 0.83 with a median of 0.63. Figure 3 shows that here in Phase 2, subjects did indeed pick up on the now discrete bimodality much better than in the previous studies.

## 7. Quantifying learning

While one of us (HP) felt that the results clearly showed that distributional shape learning was negligible except when the distribution had a gross qualitative feature (zero density in the middle region), EV felt it would still be useful to explore the extent of learning quantitatively.

To characterize learning in these experiments, we therefore ascertained which precision of a kernel density estimate applied to the training observations (*x_i_* being the position seen on a given training trial) best captured the responses produced by our observers. As the range of possible responses is bounded, we created a ‘Beta kernel’ parametrized by one precision parameter, *k:*

$${\hat{f}}_{k}(x)=\frac{1}{n}\overset{n}{\underset{i=1}{\sum }}K_{k}(x|x_{i})$$

and

$$K_{k}(x|x_{i})=\text{Beta}(x|1+x_{i}\times 10^{k},1+(1-x_{i})\times 10^{k})$$

**Figure 4.**
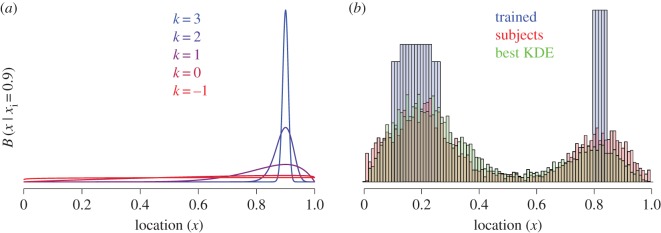
(*a*) Beta kernel for an observed value of 0.9 for different precision parameters. Kernel precision parameters less than 0 yield an effectively uniform distribution. (*b*) Results of Experiment 3: trained distribution (blue), subjects' response distribution (red), and the best-fitting (*k* = 1.09) Beta kernel density estimate of the trained distribution (green; note yellow arises where red and green overlap).

This fitting was done by obtaining the distribution over positions as the kernel density estimate (sum over all kernels for all training data, normalized), for a given *k*. Then the likelihood of a subject's responses under that distribution was calculated for each *k*. Finally, the maximum-likelihood *k* was taken as the estimate. When *k* is large (greater than 0), the kernel amounts to a beta distribution peaked at the observed value, with the distribution approaching a single spike at the observed value as *k* increases. When *k* is small (less than 0), the kernel loses just about all of the information about the observed value, and yields a uniform (0, 1) distribution (figure 4). We fit the kernel precisions parameter to individual subjects in each of our experiments, as well as the aggregate across-subject data shown in figures 1–3.

**Figure 5.**
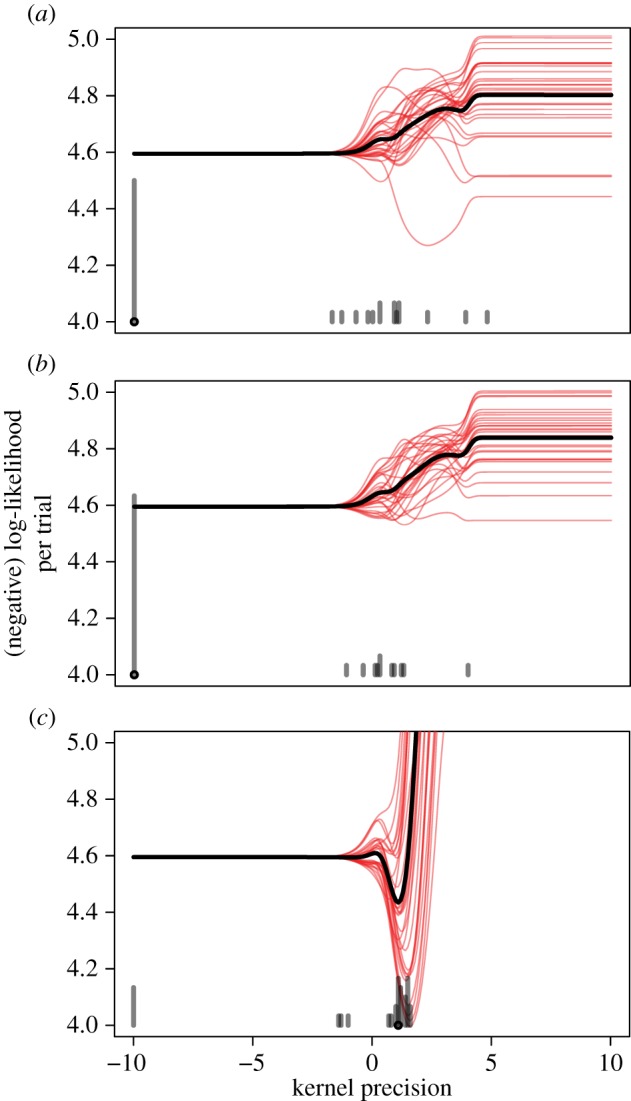
Quality of fit (*y*-axis; lower is better) of different kernel precision values (*x*-axis) for Experiments 1, 2 and 3 (from (*a*) to (*c*)). Individual subject fits are shown in red, while the fit to the aggregate data is shown in black. Grey bars at the bottom of each panel are a histogram (across subjects) of the best-fitting *k*-values (black circle indicates the best-fitting value for the aggregate over all subjects). Although a minority of subjects reveal some learning (positive kernel precision) in Experiments 1 and 2, for the most part, kernel precisions are very negative, indicating that subjects do not reliably capture any of the training distribution signal in their responses. By contrast, Experiment 3 shows reliable learning.

**Figure 6.**
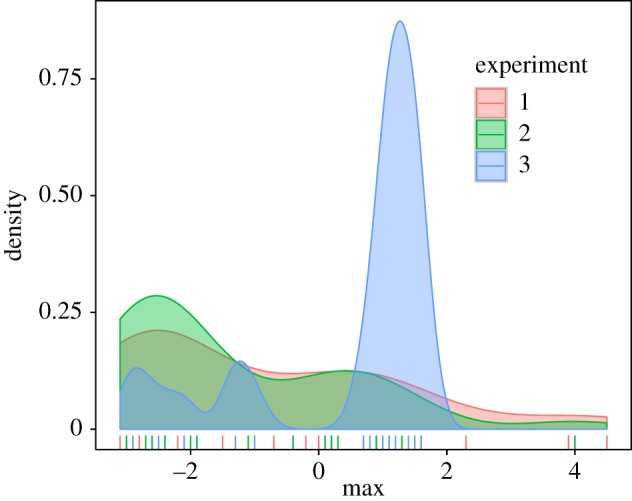
Histogram of subject MAP estimates for Experiments 1–3.

Figure 5 shows how well different values of *k* fit individual subjects in each of our experiments. Only Experiment 3 shows that subjects learned something from the training distribution—as indicated by an advantage of kernel precisions greater than 0 (23 of 30 subjects have a best-fitting *k*_ML_ > 0). By contrast, for Experiments 1 and 2, the best-fitting kernel precision is very negative for most subjects (*k*_ML_ > 0 for 10/30 and 9/30, respectively), indicating that most subjects' responses reflect effectively zero influence of the training distribution. To examine these results across experiments, we estimated maximum *a posteriori* values of *k* (with loosely informative priors of *k *∼ *N*(0, 5) to avoid indistinguishable regions for very negative values of *k*) for each subject. A one-way ANOVA on subject MAP estimates (figure 6) showed statistical significance across the three experiments, *F*_2,89_ = 8.54, *p* < 0.001.

## 8. General discussion

The results reported here show that when people are exposed to a stream of stimuli whose properties on an action-relevant dimension (here, location) conform to a bimodal distribution, they fail to spontaneously learn the bimodality. The same is true even when they are told to try to learn the distribution of locations (Experiment 2). However, when the continuous bimodal distribution was discretized by adding a zero-density gap between the two modes, people showed clear distributional knowledge (Experiment 3). We are inclined to reject the possibility that subjects learned the distribution in Experiments 1 and 2, but failed to produce under the ‘generate samples’ testing procedure because the same procedure yielded clear positive findings in Experiment 3.

The results are amenable to a number of possible interpretations, some of which we will mention here without taking any strong view (reflecting the current authors' friendly adversarial collaboration noted in the Introduction). One intriguing interpretation is that there is no general non-parametric learning of continuous probability distributions, and the results of Experiment 3 arose because the distribution could be readily discretized on account of the zero-density interval between the two modes. This would be consistent with the idea that discreteness affects the ease in which distributions can be abstracted.

Another possibility is that people have a strong tendency to learn by ‘parameter tuning’ of particular functional forms of distributions, rather than learning distributions non-parametrically—an account echoed in results suggesting that perceptual learning amounts to parameter tuning of feature relationships, rather than learning new relationships among features [15]. A more mundane but still interesting possibility is that non-parametric learning of a distribution proceeds with imperfect and incomplete memory, which renders the distributions in Experiments 1 and 2 too subtle to be learned. Although learning does occur when the subtle bimodal distribution was made more notable in Experiment 3. These findings contrast the conclusions from Acerbi *et al*. [16] where the discernibility of complex distributions does not modulate performance. However, their subjects were given explicit distribution information to be used in their spatial estimation task. This discrepancy may reflect different processes when using explicit information versus generating novel samples from distributions. Yet another possibility, attributed to a referee suggestion on an earlier version of this manuscript, might be subjects represent the distribution faithfully as changes in distance from trial-by-trial (i.e. allocentrically) rather than over the spatial width of the line. Finally, a related possibility is that people have prior assumptions strongly favouring unimodal distributions, and the data provided in Experiments 1 and 2 (perhaps corrupted by memory) are insufficient to overcome such priors.

Further support for this possibility comes from a recent study by Sanborn & Beierholm [17]. These investigators had subjects estimate the number of circles in a display (the number ranged from a minimum of 23 to at most 35). Quantities were drawn from discretized bimodal or quadrimodal distributions and feedback was provided. While Sanborn & Beierholm's bimodal distribution did not have zero density in the middle, by contrast with our Experiment 3, the two modes were always at the most extreme left and right positions within the distribution (e.g. trials with 23 and 29 circles appeared with a probability of 0.3 and trials with 24–28 circles appeared with a probability of 0.08 with a total of 700 trials). The quadrimodal distribution combined two bimodal distributions with a zero density region interposed between them (e.g. trials with 23, 25, 29 or 31 circles appeared with a probability of 0.2; trials with 24 or 30 circles appeared with a probability of 0.1; all other possible quantities of circles had a probability of 0). Subjects' behavioural responses (shown as conditional response distributions) suggested they had learned a good deal about the distributions. Given their discrete character, these findings seem consistent with the findings of Experiment 3.

## 9. Suggestions for future research

So what do these three studies tell us? It seems that learning fine-grained structure of observed probability distribution may not be as efficient as prior literature might seem to imply. The clear discrepancy between Experiments 1 and 2, and Experiment 3 suggests an intriguing possibility: only when a continuous distribution may be easily discretized do people engage in some form of non-parametric learning; otherwise, they tend to learn only a few moments of the distribution (such as the commonly investigated tendency to learn the mean and variance).

Of course, before such a hypothesis might be acknowledged, it would be important to untangle more mundane accounts: perhaps strong priors about unimodality, coupled with an imperfect memory for exemplars, is responsible for this pattern of results.

## Supplementary Material

Fixed Trial Sequences

## Data accessibility

Our data are deposited at FigShare: https://doi.org/10.6084/m9.figshare.4767997.
