# Supplementary material for: How effective is incidental learning of the shape of probability distributions?
Source: R Soc Open Sci. 2017 Aug 2;4(8):170270. doi: 10.1098/rsos.170270 (PMC5579092; doi:10.1098/rsos.170270)
Supplement: Fixed Trial Sequences [file rsos170270supp1.doc]

Randy Tran*, Ed Vul, Hal Pashler

*To whom correspondence should be addressed. E-mail: r4tran@ucsd.edu

The following 180 trials were the fixed sequence of locations used for all subjects in Experiments 1-3.

| **Trial Number** | **Experiment 1 & 2** | **Experiment 3** |
| --- | --- | --- |
| 1 | 0.57 | 0.82 |
| 2 | 0.15 | 0.14 |
| 3 | 0.16 | 0.13 |
| 4 | 0.82 | 0.23 |
| 5 | 0.80 | 0.15 |
| 6 | 0.85 | 0.83 |
| 7 | 0.44 | 0.23 |
| 8 | 0.22 | 0.82 |
| 9 | 0.84 | 0.10 |
| 10 | 0.13 | 0.12 |
| 11 | 0.11 | 0.23 |
| 12 | 0.01 | 0.19 |
| 13 | 0.82 | 0.20 |
| 14 | 0.65 | 0.18 |
| 15 | 0.22 | 0.82 |
| 16 | 0.70 | 0.83 |
| 17 | 0.40 | 0.83 |
| 18 | 0.84 | 0.14 |
| 19 | 0.51 | 0.15 |
| 20 | 0.62 | 0.81 |
| 21 | 0.05 | 0.84 |
| 22 | 0.17 | 0.19 |
| 23 | 0.26 | 0.84 |
| 24 | 0.37 | 0.13 |
| 25 | 0.24 | 0.26 |
| 26 | 0.80 | 0.80 |
| 27 | 0.83 | 0.82 |
| 28 | 0.20 | 0.25 |
| 29 | 0.27 | 0.21 |
| 30 | 0.03 | 0.13 |
| 31 | 0.12 | 0.21 |
| 32 | 0.08 | 0.11 |
| 33 | 0.83 | 0.17 |
| 34 | 0.12 | 0.81 |
| 35 | 0.18 | 0.13 |
| 36 | 0.23 | 0.84 |
| 37 | 0.20 | 0.83 |
| 38 | 0.72 | 0.10 |
| 39 | 0.84 | 0.12 |
| 40 | 0.21 | 0.80 |
| 41 | 0.25 | 0.26 |
| 42 | 0.17 | 0.16 |
| 43 | 0.06 | 0.18 |
| 44 | 0.14 | 0.81 |
| 45 | 0.54 | 0.14 |
| 46 | 0.80 | 0.25 |
| 47 | 0.10 | 0.17 |
| 48 | 0.68 | 0.81 |
| 49 | 0.23 | 0.16 |
| 50 | 0.88 | 0.14 |
| 51 | 0.96 | 0.12 |
| 52 | 0.23 | 0.20 |
| 53 | 0.21 | 0.26 |
| 54 | 0.18 | 0.25 |
| 55 | 0.77 | 0.16 |
| 56 | 0.61 | 0.25 |
| 57 | 0.12 | 0.15 |
| 58 | 0.94 | 0.81 |
| 59 | 0.64 | 0.22 |
| 60 | 0.58 | 0.80 |
| 61 | 0.50 | 0.22 |
| 62 | 0.41 | 0.18 |
| 63 | 0.24 | 0.11 |
| 64 | 0.53 | 0.20 |
| 65 | 0.14 | 0.80 |
| 66 | 0.11 | 0.84 |
| 67 | 0.09 | 0.21 |
| 68 | 0.19 | 0.13 |
| 69 | 0.83 | 0.12 |
| 70 | 0.83 | 0.16 |
| 71 | 0.14 | 0.10 |
| 72 | 0.33 | 0.16 |
| 73 | 0.49 | 0.81 |
| 74 | 0.25 | 0.22 |
| 75 | 0.84 | 0.83 |
| 76 | 0.23 | 0.84 |
| 77 | 0.42 | 0.11 |
| 78 | 0.52 | 0.16 |
| 79 | 0.15 | 0.24 |
| 80 | 0.69 | 0.14 |
| 81 | 0.02 | 0.16 |
| 82 | 0.07 | 0.84 |
| 83 | 0.48 | 0.25 |
| 84 | 0.82 | 0.22 |
| 85 | 0.81 | 0.13 |
| 86 | 0.63 | 0.26 |
| 87 | 0.74 | 0.26 |
| 88 | 0.47 | 0.18 |
| 89 | 0.83 | 0.12 |
| 90 | 0.16 | 0.17 |
| 91 | 0.20 | 0.10 |
| 92 | 0.38 | 0.18 |
| 93 | 0.13 | 0.84 |
| 94 | 0.28 | 0.24 |
| 95 | 0.16 | 0.16 |
| 96 | 0.82 | 0.12 |
| 97 | 0.81 | 0.24 |
| 98 | 0.81 | 0.24 |
| 99 | 0.82 | 0.24 |
| 100 | 0.17 | 0.26 |
| 101 | 0.26 | 0.21 |
| 102 | 0.46 | 0.83 |
| 103 | 0.10 | 0.80 |
| 104 | 0.79 | 0.10 |
| 105 | 0.20 | 0.10 |
| 106 | 0.87 | 0.17 |
| 107 | 0.93 | 0.14 |
| 108 | 0.22 | 0.15 |
| 109 | 0.83 | 0.21 |
| 110 | 0.83 | 0.23 |
| 111 | 0.95 | 0.22 |
| 112 | 0.11 | 0.80 |
| 113 | 0.98 | 0.20 |
| 114 | 0.76 | 0.19 |
| 115 | 0.92 | 0.10 |
| 116 | 0.19 | 0.24 |
| 117 | 0.16 | 0.21 |
| 118 | 0.15 | 0.22 |
| 119 | 0.13 | 0.18 |
| 120 | 0.34 | 0.80 |
| 121 | 0.80 | 0.15 |
| 122 | 0.22 | 0.83 |
| 123 | 0.30 | 0.19 |
| 124 | 0.81 | 0.20 |
| 125 | 0.99 | 0.15 |
| 126 | 0.29 | 0.18 |
| 127 | 0.71 | 0.19 |
| 128 | 0.84 | 0.82 |
| 129 | 0.32 | 0.11 |
| 130 | 0.39 | 0.83 |
| 131 | 0.45 | 0.83 |
| 132 | 0.80 | 0.13 |
| 133 | 0.18 | 0.23 |
| 134 | 0.67 | 0.20 |
| 135 | 0.26 | 0.22 |
| 136 | 0.12 | 0.81 |
| 137 | 0.81 | 0.17 |
| 138 | 0.82 | 0.21 |
| 139 | 0.75 | 0.23 |
| 140 | 0.84 | 0.18 |
| 141 | 0.13 | 0.82 |
| 142 | 0.55 | 0.17 |
| 143 | 0.89 | 0.23 |
| 144 | 0.31 | 0.81 |
| 145 | 0.25 | 0.19 |
| 146 | 0.36 | 0.11 |
| 147 | 0.56 | 0.23 |
| 148 | 0.19 | 0.20 |
| 149 | 0.11 | 0.81 |
| 150 | 0.80 | 0.80 |
| 151 | 0.81 | 0.15 |
| 152 | 0.19 | 0.19 |
| 153 | 0.90 | 0.80 |
| 154 | 0.78 | 0.11 |
| 155 | 0.21 | 0.19 |
| 156 | 0.86 | 0.80 |
| 157 | 0.43 | 0.82 |
| 158 | 0.84 | 0.17 |
| 159 | 0.24 | 0.84 |
| 160 | 0.17 | 0.22 |
| 161 | 0.35 | 0.82 |
| 162 | 0.80 | 0.21 |
| 163 | 0.91 | 0.20 |
| 164 | 0.10 | 0.17 |
| 165 | 0.15 | 0.11 |
| 166 | 0.73 | 0.26 |
| 167 | 0.04 | 0.14 |
| 168 | 0.21 | 0.84 |
| 169 | 0.25 | 0.25 |
| 170 | 0.81 | 0.84 |
| 171 | 0.60 | 0.82 |
| 172 | 0.24 | 0.82 |
| 173 | 0.10 | 0.24 |
| 174 | 0.26 | 0.15 |
| 175 | 0.59 | 0.81 |
| 176 | 0.66 | 0.12 |
| 177 | 0.14 | 0.83 |
| 178 | 0.82 | 0.13 |
| 179 | 0.18 | 0.14 |
| 180 | 0.97 | 0.25 |
